# Aging Aggravates Cachexia in Tumor-Bearing Mice

**DOI:** 10.3390/cancers14010090

**Published:** 2021-12-24

**Authors:** Julia Geppert, Alina A. Walth, Raúl Terrón Expósito, Doris Kaltenecker, Pauline Morigny, Juliano Machado, Maike Becker, Estefania Simoes, Joanna D. C. C. Lima, Carolin Daniel, Mauricio Berriel Diaz, Stephan Herzig, Marilia Seelaender, Maria Rohm

**Affiliations:** 1 Institute for Diabetes and Cancer, Helmholtz Center Munich, 85764 Neuherberg, Germany; julia.zuber@helmholtz-muenchen.de (J.G.); Alina.walth@helmholtz-muenchen.de (A.A.W.); raul.terron@helmholtz-muenchen.de (R.T.E.); doris.kaltenecker@helmholtz-muenchen.de (D.K.); pauline.morigny@helmholtz-muenchen.de (P.M.); juliano.machado@helmholtz-muenchen.de (J.M.); estefania.simoes@helmholtz-muenchen.de (E.S.); mauricio.berrieldiaz@helmholtz-muenchen.de (M.B.D.); stephan.herzig@helmholtz-muenchen.de (S.H.); 2 Joint Heidelberg-IDC Translational Diabetes Program, Inner Medicine 1, Heidelberg University Hospital, 69120 Heidelberg, Germany; 3 German Center for Diabetes Research (DZD), 85764 Neuherberg, Germany; maike.becker@helmholtz-muenchen.de (M.B.); carolin.daniel@helmholtz-muenchen.de (C.D.); 4 Institute for Diabetes Research, Research Group Immune Tolerance in Diabetes, Helmholtz Diabetes Center at Helmholtz Center Munich, 85764 Neuherberg, Germany; 5 Department of Surgery and LIM 26, Faculdade de Medicina, University of Sao Paulo, Sao Paulo 01246-903, Brazil; joanna.carolacorreialima@ndm.ox.ac.uk (J.D.C.C.L.); seelaender@usp.br (M.S.); 6 Division of Clinical Pharmacology, Department of Medicine IV, Ludwig-Maximilians-Universität, 80539 Munich, Germany; 7 Chair Molecular Metabolic Control, TUM School of Medicine, Faculty of Medicine, Technical University Munich, 80333 Munich, Germany

**Keywords:** aging, cachexia, cancer, mouse models

## Abstract

**Simple Summary:**

Cachexia is a deadly disease that accompanies many different types of cancers. Animal studies on cachexia have so far mostly been conducted using young mice, while cancer in humans is a disease of high age. Mouse models used to date may therefore not be suitable to study cachexia with relevance to patients. By comparing young and old mice of three different strains and two different tumor types, we here show that the age of mice has a substantial effect on cachexia progression (specifically body weight, tissue weight, fiber size, molecular markers) that is dependent on the mouse strain studied. This is independent of glucose tolerance. The cachexia markers IL6 and GDF15 differ between ages in both mice and patients. Future studies on cachexia should consider the age and strain of mice.

**Abstract:**

Background: Cancer is primarily a disease of high age in humans, yet most mouse studies on cancer cachexia are conducted using young adolescent mice. Given that metabolism and muscle function change with age, we hypothesized that aging may affect cachexia progression in mouse models. Methods: We compare tumor and cachexia development in young and old mice of three different strains (C57BL/6J, C57BL/6N, BALB/c) and with two different tumor cell lines (Lewis Lung Cancer, Colon26). Tumor size, body and organ weights, fiber cross-sectional area, circulating cachexia biomarkers, and molecular markers of muscle atrophy and adipose tissue wasting are shown. We correlate inflammatory markers and body weight dependent on age in patients with cancer. Results: We note fundamental differences between mouse strains. Aging aggravates weight loss in LLC-injected C57BL/6J mice, drives it in C57BL/6N mice, and does not influence weight loss in C26-injected BALB/c mice. Glucose tolerance is unchanged in cachectic young and old mice. The stress marker GDF15 is elevated in cachectic BALB/c mice independent of age and increased in old C57BL/6N and J mice. Inflammatory markers correlate significantly with weight loss only in young mice and patients. Conclusions: Aging affects cachexia development and progression in mice in a strain-dependent manner and influences the inflammatory profile in both mice and patients. Age is an important factor to consider for future cachexia studies.

## 1. Introduction

Cachexia affects a large percentage of patients with cancer. It is defined by involuntary weight loss and loss of muscle and adipose tissue mass, leading to reduced quality of life, frailty, worsened treatment outcomes, and often ultimately death [1]. Currently, there is no treatment available, which effectively improves both weight loss and functional muscle impairment. As cancer incidence rises dramatically with age [2], it is to be expected that the same is true for cachexia. Indeed, cachexia is more prevalent in cancers of older age, such as gastric cancer [3]. However, systematic studies investigating cachexia in aged individuals are still scarce to date.

Mouse models represent important tools for cachexia research, as they capture some of the systemic effects of body wasting, while still being easy to manipulate pharmacologically and genetically. Next to genetic models such as APC min [4] or KPC [5], the Colon26 (C26) and Lewis Lung Cancer (LLC) models [6,7] are very commonly used mouse models of cancer cachexia wherein tumor cells are implanted subcutaneously into syngeneic mice, causing tumor growth and cancer cachexia within 2–3 weeks. Studies with genetic cachexia, but also implantation models, have largely used adolescent or young adult mice (typically 6–14 weeks of age) so far. This relates to 15–25 year old humans [8]—an age in which cancer is still rare. Recent studies have argued that cancer research needs to address the age status of experimental animals much better in order to increase the rigor of the research results [8,9]. Thus, in order to better account for the largely aged population of patients with cachexia, we hypothesized that aged mice would be better suited to study cachexia, as underlying conditions such as mild sarcopenia, glucose intolerance, or altered immune function may influence cachexia development also in mice.

The C57BL/6 mouse line is the most commonly used line in animal studies, and most knockout models were created using this line. In recent years, it has become increasingly evident that there is no such thing as the one C57BL/6 mouse, but that indeed C57BL/6J and C57BL/6N mice have considerable phenotypic and genotypic differences. Initially established at the Jackson Laboratory, the C57BL/6J mice were passed on to the National Institutes of Health (NIH) in 1951, leading to the C57BL/6N line. They have now been separated for >200 generations, leading to ~200 single nucleotide polymorphisms, ~500 structural variants, and 27 phenotype parameters, including insulin sensitivity and body composition [10] which are most relevant for cachexia development. LLC cells originated from the original C57BL/6 line in 1951 [11]; hence, they grow in both J and N mice. Both have been used for cachexia studies. Despite the known phenotypic differences between the C57BL/6 substrains, many studies do not assess or report the substrain used for their experiments.

Using LLC injection into C57BL/6J and C57BL/6N mice and C26 cell injection into BALB/c mice, we compared cachexia development in young adult (aged 2–4 months) and aged (15–20 months) mice (relating to approximately 60–75 years of age in humans, [8]). We found that LLC tumors induced body weight loss only in C57BL/6J, but not C57BL/6N, mice of young age, and produced a much stronger wasting phenotype in older animals. Likewise, the LLC-driven increases in markers of muscle atrophy and adipose tissue inflammation and mitochondrial stress were potentiated by higher age. Muscle fiber size decreased in all cachexia models. Circulating levels of interleukin 6 (IL6) were not a reliable indicator of cachexia in all mouse models as IL6 elevation seemed specific to the C26 model. C26-cell induced cachexia was independent of age in terms of body weight loss, fiber size, and molecular cachexia markers. In patients, we observed significant correlations between inflammation and body weight loss only in the young population. Our study therefore suggests that age is an important and so far underrepresented determinant of cachexia in mice, and likely also in patients.

## 2. Materials and Methods

### 2.1. Mouse Models

Mouse studies were performed using male BALB/cAnNCrl, C57BL/6J, or C57BL6/N mice of two different age groups. All mice were obtained from Charles River Laboratories (CRL, Brussels, Belgium) and aged in-house. Experiments were initiated when young adult mice had reached an age of 2–4 months, and aged mice an age of 15–20 months. All mice were maintained under specific pathogen-free conditions on a 12 h light–dark cycle at 22 °C with ad libitum access to regular rodent chow diet (Kliba Nafag #3437, Provimi Kliba AG, Switzerland; Kaiseraugst, Switzerland) and water. In each animal experiment, mice were assigned to groups in a manner that body weight was similar between groups of the same age as confirmed by non-significant statistical analysis. Animal handling and experimentation were performed in accordance with the institutional animal welfare officer, and the necessary licenses were obtained from the state ethics committee and government of Upper Bavaria (nos. ROB-55.2-2532.Vet_02-16-136 and ROB-55.2-2532.Vet_02-18-93). The number of mice used for each experiment is indicated in the figure legends. For glucose and insulin tolerance tests (GTT and ITT, respectively), mice were fasted for 6 h before intraperitoneal injections (i.p.). For GTTs, fasted mice were i.p. injected with 2 g D-glucose per kg body weight. For ITTs fasted mice were i.p. injected with 0.8 U insulin per kg body weight. In different experiments, mice were injected subcutaneously into the right flank with 2 million Lewis lung cancer (LLC), or 1 million C26 cells, resuspended in 50 µL of Dulbecco’s phosphate-buffered saline (DPBS, Thermo Fisher, # 14190250, Waltham, MA, USA). Non-tumor, healthy control mice were injected with 50 μL of DPBS. Mice were monitored for 2–4 weeks after tumor cell implantation, and the tumor growth, body weight, and food intake were recorded daily. Tumor growth was monitored by caliper measurement, and tumor volume was calculated using the formula V = π/6 × 1.69 × (L × W)^3/2^ [12], where L is the largest tumor diameter and W is the perpendicular tumor diameter. Mice were sacrificed by an overdose of ketamine/xylazine when one or more of four termination criteria were fulfilled: time after injection > 28 days, ulceration, tumor diameter > 1.5 cm, or cachexia as defined by body weight loss > 15% or body condition score (BCS) < 2 [13]. The BC scoring technique was used to assess the overall health status of the mice during the course of the experiment as the growing tumors can mask body weight changes due to extensive growth. To apply the BCS score, the amount of flesh covering bony protuberances of the mice was monitored and transformed into a score ranging from 1 to 5. While mice with a BCS = 5 are defined as obese, mice with a BCS = 1 are underconditioned and emaciated. A mouse with a BCS = 3 was categorized as being in an optimal condition, whereas mice with a BCS = 2 are already underconditioned with segmentation of the vertebral column and distinct dorsal pelvic bones.

Blood was withdrawn from the vena cava, transferred into EDTA tubes (Kabe Labortechnik, #78001) and centrifuged at 2000× *g*, 4 °C for 5 min. Plasma was divided into 50 μL aliquots, immediately snap frozen in liquid nitrogen and stored at −80 °C until further processing. Tumors and organs including epididymal and inguinal white adipose tissue (WAT), and gastrocnemius (GC) muscles were collected, snap frozen, and stored at −80 °C.

### 2.2. Patient Cohort and Samples

Enrolment of Brazilian gastrointestinal cancer patients (stages I–IV) occurred at the Surgical Clinic of the University Hospital of São Paulo after signature of the fully informed consent. This study was approved by the Ethics Committee on Research Involving Human Subjects of the University of the São Paulo Biomedical Sciences Institute (CEP 1151/13 CAAE n 5493116.6.0000467) and by the University Hospital (CEP1390/14 CAAE n 54930116.6.3001.0076) and is in accordance with the Declaration of Helsinki. Exclusion criteria: BMI > 29.9 kg/m^2^, chronic anti-inflammatory therapy or chronic inflammatory processes not related to cachexia, chemotherapy treatment (at the time or recent past 5 years), AIDS, or liver or kidney failure. Approximately 20 mL of blood were collected in pre-surgical fast at the hospital. Biochemical analysis was performed with automatic LABMAX 240^®^ equipment (Labtest, Lagoa Santa, Brazil) using commercial kits. The hemoglobin concentration was obtained from the hospital records before the surgery. Cytokines were analyzed by Luminex^®^xMAP™ (Bio-Rad Laboratories, Hercules, CA, USA) technology and performed according to the manufacture’s protocol (Human Cytokine/Chemokine Magnetic Bead Panel Cat. #HCYTMAG-60K-PX30) on a MAGPIX^®^ instrument (Life Technologies, Carlsbad, CA, USA). Proteins were quantified with the equipment-specific software (xPONENT^®^ 4.2, Life Technologies, Carlsbad, CA, USA). Human GDF15 plasma levels were assessed using a specific ELISA kit (R&D Systems # DGD150, Minneapolis, MN, USA) according to the manufacturer’s instructions. Cancer patients were classified as weight stable (Ws) or cachectic (Cx), following Evans et al. [14].

### 2.3. Plasma Analysis and Enzyme-Linked Immunosorbent Assays

Total cholesterol, LDL, glucose, NEFA, albumin, total proteins, and triacylglycerol (TAG) plasma levels of mice were measured using a Beckman Coulter AU480 Chemistry Analyzer (Beckman Coulter, Brea, CA, USA).

Murine plasma levels of IL6 and GDF15 were assessed using specific ELISA kits (R&D Systems #M6000B and #MGD150, Minneapolis, MN, USA) according to the manufacturer’s instructions. Dilution of plasma samples for mouse GDF15 and IL6 detection was optimized to 1/2 and 2/5, respectively.

### 2.4. RNA Extraction and Reverse Transcription

For total RNA extraction, frozen organ pieces were homogenized in TRIzol (Life Technologies, # 15596018, Carlsbad, CA, USA) using Qiagen’s TissueLyser II (Qiagen, # 85300, Hilden, Germany. RNA was then isolated by adding chloroform, precipitated with isopropanol, washed 3 times with 75% ethanol and finally resuspended in RNase/DNase free water. RNA concentration was quantified using a NanoDrop2000 spectrophotometer (Thermo Fisher, # ND-2000, Waltham, MA, USA) and 1 µg of total RNA was treated with DNase (Life Technologies, # 18068015, Carlsbad, CA, USA) and reverse-transcribed into cDNA (Life Technologies, # 4368814, Carlsbad, CA, USA) according to the manufacturer’s protocol.

### 2.5. Quantitative PCR

Real-time quantitative PCR was conducted using Applied Biosystems’ QuantStudio 6 or 7 Flex Real-Time PCR System (Applied Biosystems, # 4485691, 4485695, Waltham, MA, USA) and Takyon™ Low Rox Probe MasterMix dTTP Blue (Eurogentec, # UF-LPMT-B0710, Seraing, Belgium). RNA expression data were quantified according to the ΔCt method [15], and normalized to levels of TATA-box binding protein RNA (Tbp, Mm01277042_m1). The following Taqman probes (Applied Biosystems) were used: Trim63 [Mm01185221_m1], Fbxo32 [Mm00499523_m1], Foxo3a [Mm01185722_m1], Ctsl [Mm00515597_m1], Cdkn1a [Mm00432448_m1], Atgl [Mm00503040_m1], Plin4 [Mm00491061_m1], Nrf1 [Mm00447996_m1], Nrf2 [Mm00477784_m1], Opa1 [Mm00453879_m1], Tfam [Mm00447485_m1], Atg12 [Mm00503201_m1], p62 [Mm00448091_m1], Atg4 [Mm04214755_s1], Atg5 [Mm01187303_m1], Ddit3 [Mm00492097_m1], Xbp1 [Mm00457357_m1], Gadd34 [Mm00435119_m1], Atf3 [Mm00476032_m1], Hspa5 [Mm00517691_m1], Nos2 [Mm00440502_m1], Sod2 [Mm01313000_m1], Ddb1 [Mm00497159_m1], Casp3 [Mm01195085_m1], Casp9 [Mm00516563_m1], Bcl2 [Mm00477631_m1], Bnip3 [Mm01275600_g1], Mff [Mm01273401_m1] Pgc1 [Mm01208835_m1], IL6 [Mm00446190_m1], IL1b [Mm00434228_m1], Tnfa [Mm00443258_m1], Ccl2 [Mm00441242_m1], Gdf15 [Mm00442228_m1], Hsl [Mm00495359_m1], Mgl [Mm00449274_m1], Cpt1a [Mm00550438_m1].

### 2.6. Immunohistochemistry

GC muscles for histology were snap frozen in liquid nitrogen. Cryosections of skeletal muscle cell membrane were stained for Wheat Germ agglutinin, Alexa Fluor™ 488 Conjugate (W11261—Thermo Fisher Scientific, Waltham, MA, USA) and DAPI and were examined on a Nikon Eclipse Ci Upright Microscope (×20 magnification). For each animal, the cross-sectional area (CSA) of more than 500 fibers from 5 different sites within a consistent region of the GCmuscle was quantified using the software ImageJ/Fiji (version 1.51).

### 2.7. Protein Extraction and Immunoblotting

Proteins were extracted from frozen gastrocnemius muscle following lysis in ice-cold lysis buffer (50 mM Tris/HCl pH 7.6, 150 mM NaCl, 1% Triton X-100, 0.5% Sodiumdeoxycholate, 0.1% SDS, 1 X cOmplete protease inhibitor cocktail from Roche, 1X PhosSTOP cocktail from Roche). Measures of 30 μg of protein extracts were separated on 8–16% tris-glycine gels, blotted onto nitrocellulose membrane, and incubated with the following primary antibodies: Vinculin (1/10,000, abcam #ab129002, Cambridge, UK), AKT (1/1000, Cell Signaling #9272, Danvers, MA, USA), pS473-AKT (1/1000, Cell Signaling #4060, Danvers, MA, USA), GSK3β (1/1000, Cell Signaling #9315, Danvers, MA, USA), pS9-GSK3β (1/1000, Cell Signaling #5558, Danvers, MA, USA). Anti-rabbit or anti-mouse IgG coupled to horseradish peroxidase were used as secondary antibodies, and immunoreactive proteins were determined by chemiluminescence using a ChemiDoc MP System (Bio-Rad Laboratories, Hercules, CA, USA). All the whole western blot figures can be found in the Supplementary Materials.

### 2.8. Cell Staining for Flow Cytometry

The following antibodies were used for flow cytometry (reactivity, fluorochrome, clone, manufacturer): CD11b, Pacific Blue, M1/70, BioLegend; CD11c Brilliant Violet 421, N418, BioLegend; B220 Pacific Blue, RA3-6B2, BioLegend; CD14, V450, rmC5-3, BD Biosciences; F4/80, Pacific Blue, BM8, BioLegend; CD8a, Brilliant Violet 605, 53-6.7, BioLegend; CD4, Alexa Fluor 700, RM4-5, BioLegend; FoxP3, FITC, FJK-16s, eBioscience.

Unspecific binding of antibodies was prevented by incubation of cell suspensions with Fc-Block (TruStain FcX™ anti-mouse CD16/32 Antibody, 93, BioLegend) for 10 min on ice, followed by Flow cytometric staining for 30 min on ice in the dark. To detect intracellular protein expression, T cells were fixed and permeabilized using the Foxp3 Staining Buffer Set (eBioscience, San Diego, CA, USA) after surface staining as recommended by the manufacturer. Cells were passed through a 40 μm cell strainer (NeoLab, Heidelberg, Germany) to remove large debris.

Cells were acquired on a BD FACSAriaIII flow cytometer using FACSDiva software V6.1.3 with optimal compensation and gain settings based on unstained cells and single-color stained beads. Doublets were excluded based on SSC-A vs. SSC-W and FSC-A vs. FSC-W plots. Fixed cell populations were gated on the basis of cell side and forward scatter. Cells positive for Fixable Viability Dye eFluor450 (eBioscience) were excluded. Samples were analyzed using FlowJo software v10.8.0 (TreeStar Inc., Ashland, OR, USA).

### 2.9. Statistical Analysis

Results from biological replicates were expressed as mean ± standard error of the mean (s.e.m.). Statistical analysis was performed using GraphPad Prism 9.2. Normality was tested using D’Agostino-Pearson and Shapiro–Wilk normality tests. Statistical tests were two-sided. Unpaired Student’s *t*-tests or Mann-Whitney tests were performed to compare two conditions. Paired two-way analysis of variance (ANOVA) with Tukey’s and Bonferroni post hoc tests were used to compare two variables. Linear regression analysis was performed to test association between two variables. Log-rank (Mantel-Cox) test was used to compare time curves of cachexia development between groups.

## 3. Results

### 3.1. Tumor Growth Is Largely Unaffected by Age

**Figure 1:**
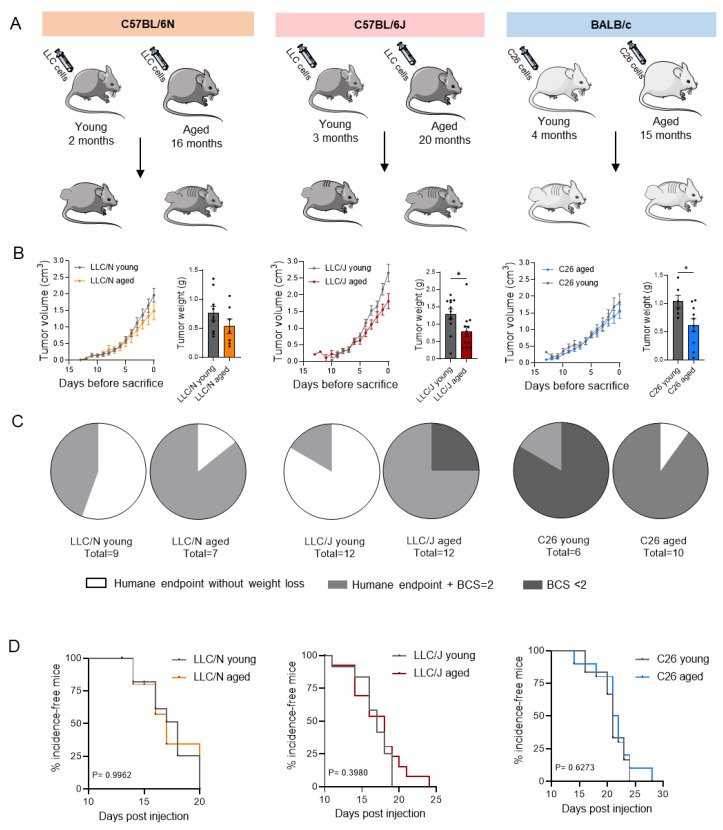
**Tumor growth is largely unaffected by age in different mouse models with various tumor entities.** (**A**) Diagram presenting three different cachexia mouse models including groups of several ages. Mice were injected with different tumor cell lines (LLC and C26). C57BL/6N (orange)—comparison of young adult (2 months, grey, *n* = 9) and aged (16 months, orange, *n* = 7) LLC-injected C57BL/6N mice. C57BL/6J (red—comparison of young adult (3 months, grey, *n* = 12) and aged (20 months, red, *n* = 12) LLC-injected C57BL/6J mice. BALB/c (blue)—comparison of young adult (4 months, grey, *n* = 6) and aged (15 months, blue, *n* = 10) C26-injected BALB/c mice. (**B**) Tumor growth over time and tumor weight at the end of the experiment in young and aged LLC/N, LLC/J, and C26-BALB/c mice. (**C**) Pie charts representing the primary termination criteria of tumor-bearing mice. We differentiated between animals that reached the humane endpoint but had a stable bodyweight (white) versus mice that reached the humane endpoint and simultaneously had already lost between 3–10 % of body weight (BCS = 2, light grey). Animals with a BCS < 2 were terminated due to cachexia. (**D**) Incidence-free time between young and aged tumor cell-injected LLC/N, LLC/J, and C26-BALB/c mice. Data are mean ± s.e.m. Statistical analyses were performed using unpaired *t*-test or Mann–Whitney test (**B**), and log-rank (Mantel–Cox) test (**D**). * *p* < 0.05.

Using three independent mouse models, we compared young adult (2–4 months) and aged (15–20 months) mice with tumors in terms of cachexia development (Figure 1). We also characterized the mice depending on strain and substrain, as fundamental differences in metabolism and behavior have previously been noted [10]. C57BL/6N and C57BL/6J mice were injected with LLC cells (mice referred to as LLC/N and LLC/J), BALB/c mice were injected with C26 cells (Figure 1A). Tumors were palpable after ~7 days in all groups and grew to a similar size over the course of the experiment, with younger mice showing slightly but significantly larger tumors at the end of the experiment (Figure 1B), in line with previous studies [9]. Mice were sacrificed when one or more of four termination criteria were fulfilled: time after injection > 28 days, ulceration, tumor diameter > 1.5 cm, or cachexia as defined by body weight loss > 15% or body condition score (BCS) < 2. The BCS technique was used to assess the overall health status of the mice during the course of the experiment. In LLC/N and LLC/J mice, the majority of aged tumor bearing animals developed body weight loss (BCS ≤ 2), while young animals had to be terminated mostly for other reasons (ulceration or tumor size, dependent on the model). In the C26 model, the percentage of cachectic animals (BCS ≤ 2) was largely identical in the two groups of different age (Figure 1C). Incidence-free time (a proxy for survival time) was unaltered between young and old mice (Figure 1D).

### 3.2. Strain-Dependent Differences in Cancer-Induced Loss of Body and Tissue Weights

**Figure 2:**
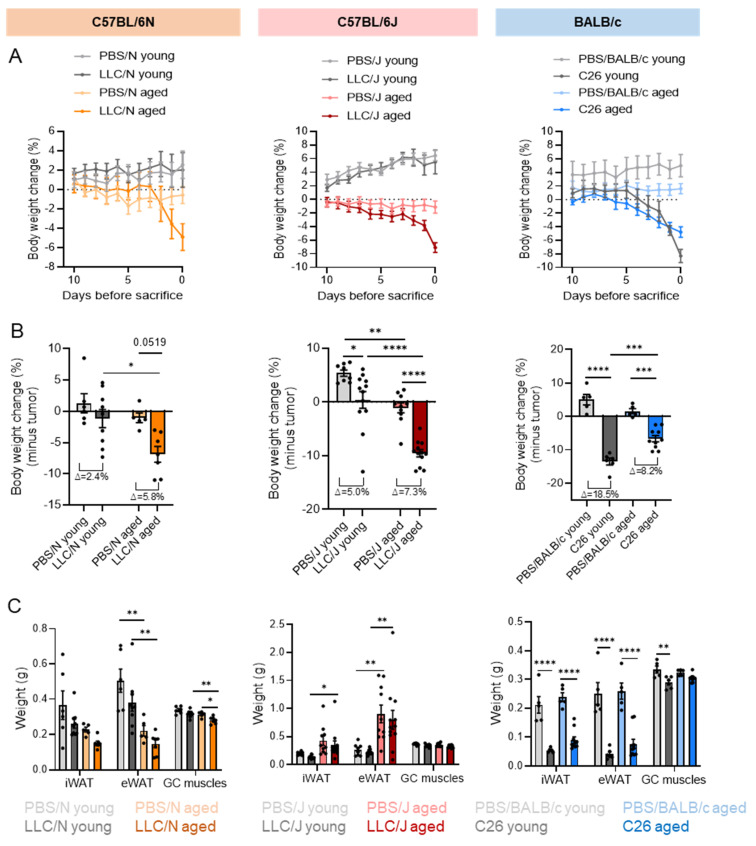
**Strain-dependent differences in cancer-induced weight loss development.** (**A**–**C**) Mice of different ages were injected with either PBS (control) or a tumor cell line (LLC or C26). Young adult mice (grey lines and bars) injected with PBS are depicted in light grey (PBS/N young *n* = 6, PBS/J young *n* = 8, PBS/BALB/c young *n* = 5), young mice injected with a tumor cell line are shown in dark grey (LLC/N young *n* = 9, LLC/J young *n* = 12, C26 young *n* = 6). Aged mice injected with PBS are depicted in light colors (PBS/N aged *n* = 5-light orange, PBS/J aged *n* = 10-light red, PBS/BALB/c aged *n* = 5-light blue), while aged mice injected with a tumor cell line are shown in dark colors (LLC/N aged *n* = 7-dark orange, LLC/J aged *n* = 12-dark red, C26 aged *n* = 10-dark blue). (**A**) Time course of body weight development (change in percentage compared to initial body weight before injection). (**B**) Change of body weight minus tumor compared to initial mass (expressed as percentage). (**C**) Tissue weights of inguinal white adipose tissue (iWAT), epididymal WAT (eWAT), and gastrocnemius skeletal muscle (GC). Data are mean ± s.e.m. Statistical analyses were performed using two-way analysis of variance (ANOVA) with Tukey’s multiple-comparison post hoc test. * *p* < 0.05, ** *p* < 0.01, *** *p* < 0.001, **** *p* < 0.0001.

A time course of body weight development showed that PBS injected young mice of all three strains still gained weight over the course of the experiment, whereas old PBS-injected mice remained weight stable (Figure 2A). There was no difference in the initial body weight between PBS and tumor cell-injected mice, but aged mice were significantly heavier than young animals, as expected (Supplemental Figure S1). Figure 2A shows the body weight change in the 10 days prior to sacrifice to account for the differences in time to humane endpoint (Figure 1D). All aged tumor cell injected mice showed severe body weight loss over the course of the experiment, starting 4 to 7 days prior to sacrifice, depending on the mouse strain (Figure 2A,B). Old LLC/J mice lost body weight earliest, and strongest, compared to the other strains, but weight loss was apparent in all aged tumor cell injected groups. In contrast, in the young age group, only C26 mice dropped body weight during the course of the experiment (Figure 2A,B). Consequently, in the young age group, tumor-subtracted body weight was only significantly reduced in the C26 model (Supplemental Figure S1B). Of note, LLC tumors induced body weight loss in some, but not all young C57BL/6J and C57BL/6N mice, with very high variability and a high rate of non-responders. LLC/J mice had a higher degree of weight loss than LLC/N mice (∆ body weight 7.3% vs. 5.8% in aged and 5.0% vs. 2.4% in young mice)—an effect we have previously anecdotally observed but which has not been systematically studied to date. Aging induced weight loss in LLC/N mice and aggravated it in LLC/J mice, while not affecting weight loss in the C26 model (Figure 2B). Likewise, epididymal and inguinal adipose tissue and gastrocnemius muscle weights were reduced by the presence of C26 tumors largely independent of age. Tissue weights were not significantly altered by the presence of LLC tumors, with the exception of aged LLC/N, highlighting a mouse strain and tumor-type-dependent response (Figure 2C).

**Figure 3:**
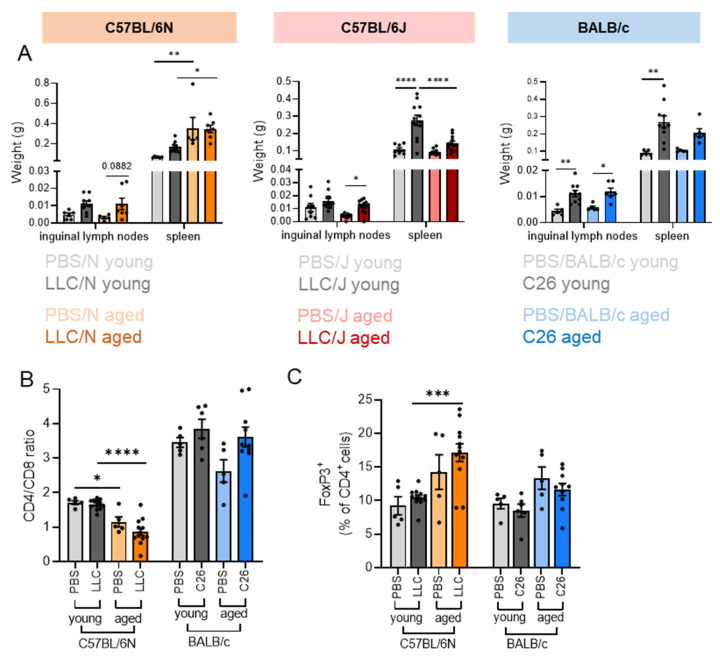
**Cachexia development in aged mice is not associated with alterations in immune cell subtype composition.** (**A**–**C**) Same mice as in Figure 2 were used for the analysis of immunologic changes upon aging. (**A**) Tissue weights of inguinal lymph nodes and spleen. (**B**) CD4/CD8 ratio of T cells isolated from blood. (**C**) Percentage of FoxP3-positive cells (% of CD4^+^ T cells). T cells isolated from blood. Data are mean ± s.e.m. Statistical analyses were performed using two-way ANOVA with Tukey’s multiple-comparison post hoc test. * *p* < 0.05, ** *p* < 0.01, *** *p* < 0.001, **** *p* < 0.0001.

Lymph nodes were enlarged in the presence of the tumors in both young and old animals, whereas spleen enlargement in tumor-bearing mice was more pronounced in young compared to aged mice (Figure 3A). T cells are instrumental in fighting cancer, and a low CD4/CD8 T cell ratio is associated with aging, which is accelerated upon various stressors [16]. Therefore, we tested the CD4/CD8 T cell ratio in blood by flow cytometry (Supplemental Figure S2). BALB/c mice had a much higher CD4/CD8 T cell ratio than C57BL/6N mice (Figure 3B), as previously reported [17]. LLC/N mice showed a reduced CD4/CD8 ratio in the aged group, but this was not affected by the presence of the tumor. The CD4/CD8 ratio was unaffected by age or tumor in BALB/c mice (Figure 3B). FoxP3^+^ regulatory T cells were elevated in aged animals (Figure 3C) as described [18], but independent of the strain. Cachexia did not cause alterations in circulating FoxP3^+^ cells (Figure 3C). Age-dependent alterations to immune cell subtypes are therefore unlikely to drive differences in cachexia development in the different mouse models.

### 3.3. Aging Increases the Tumor-Induced Expression of Atrogenes in Cachectic Muscle

**Figure 4:**
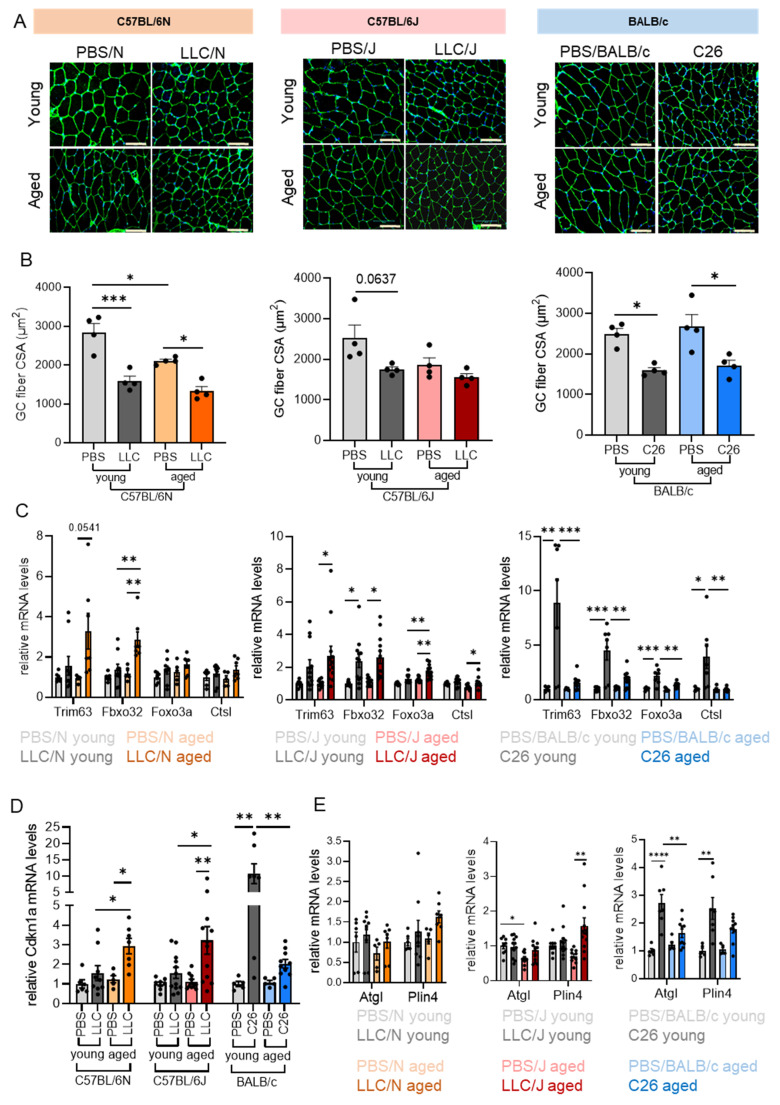
**Aging increases the tumor-induced expression of atrogenes in cachectic muscle.** (**A**) Representative staining for wheat germ agglutinin (green) and nuclei (blue) showing morphological changes in gastrocnemius muscles of PBS, LLC, and C26-injected young and aged mice; 20x magnification, scale bar 100 µm. (**B**) Quantification of fiber cross-sectional area in gastrocnemius muscles (*n* = 4, >500 fibers/mouse). (**C**–**E**) Expression of different cachexia-associated genes in gastrocnemius muscle (GC) of the same mice as in Figure 2. (**C**) mRNA expression of atrogenes in aged and young mice. (**D**) Cyclin-dependent kinase inhibitor 1A (Cdkn1a) mRNA levels. (**E**) Adipocyte triglyceride lipase (Atgl) and perilipin 4 (Plin4) gene expression. Data are mean ± s.e.m. Statistical analyses were performed using two-way ANOVA with Tukey’s multiple-comparison post hoc test. * *p* < 0.05, ** *p* < 0.01, *** *p* < 0.001, **** *p* < 0.0001.

To further characterize the cachectic phenotype in dependence of strain and age, we measured the muscle fiber cross-sectional area of the gastrocnemius muscles. Despite the apparent absence of GC mass loss in the LLC/J and young LLC/N mice (Figure 2C), we observed a reduction in fiber cross-sectional area in the presence of the tumors (Figure 4A,B), as well as upon aging. In the C26 model, the fiber size was reduced in the presence of the tumor independent of age (Figure 4A,B). On the molecular level, cachexia is often determined through the assessment of muscle atrogenes, most prominently Atrogin 1 (F-Box Protein 32/Fbxo32) and MuRF1 (Muscle-Specific RING Finger Protein 1, also called Tripartite Motif Containing 63/Trim63) [19]. To determine if aging affects molecular determinants of muscle wasting in our models, we measured the expression of the atrogenes Fbxo32, Trim63, Foxo3a, and Cathepsin-L in skeletal muscle of young and old LLC and C26 tumor cell injected mice. In line with a previous report [20], the expression of atrogenes was strongly induced in young C26 tumor cell injected mice, and to a lesser extent in aged animals (Figure 4C), also reflecting less weight loss in old vs. young BALB/c mice. Strikingly, LLC cells induced atrogene expression only in the GC muscle of aged LLC/N animals and produced a more significant increase in atrogene expression in aged compared to young LLC/J mice (Figure 4C), again reflecting cachexia severity as assessed by body weight loss (Figure 2B). Interestingly, even mild but non-significant alterations in atrogene expression as in young LLC/N and LLC/J mice were associated with reduced cross-sectional area (Figure 4A,B). Cdkn1a (Cyclin Dependent Kinase Inhibitor 1A, or p21) is an inhibitor of protein synthesis and anabolic signaling, stimulates protein breakdown, and can induce skeletal muscle atrophy [21]. In line with an important role of this factor for the cachectic muscle, we observed increased Cdkn1a expression in animals where weight loss was most apparent: aged LLC/N, aged LLC/J, and young C26 mice (Figure 4D). Mitochondrial dysfunction, autophagy, endoplasmic reticulum (ER) stress, oxidative stress, and apoptosis pathways have all previously been associated with cachexia or aging. Indeed, we have also observed tumor-induced increases in the expression of Nrf2 (nuclear factor erythroid 2-related factor 2), p62 (Sequestosome 1), Gadd34 (Ppp1r15a gene: protein phosphatase 1 regulatory subunit 15A), and Bnip3 (BCL2 interacting protein 3), which were, however, inconsistent between models (Supplemental Figure S3). In addition, several genes were decreased in the presence of the tumors, such as Tfam (Transcription factor A, mitochondrial), Atg4 (Autophagy related 4), and Atg5. Gene expression of Nos2 (Nitric oxide synthase 2), which has previously been described as important regulator of both age-related sarcopenia and cachexia [22], was significantly decreased by the presence of a tumor in the GC muscle of all models (Supplemental Figure S3). Interestingly, body weight loss was associated with a significantly increased expression of adipocyte triglyceride lipase (Atgl) and perilipin 4 (Plin4) in the muscle of C26 mice (Figure 4E). Plin4 levels were also elevated in aged LLC/J mice (Figure 4E), a sign of mild myosteatosis. This is in line with a recent meta-analysis showing increased myosteatosis as negative prognostic marker for colon cancer [23].

### 3.4. Age-Dependent Mitochondrial Stress and Inflammation in Adipose Tissue

**Figure 5:**
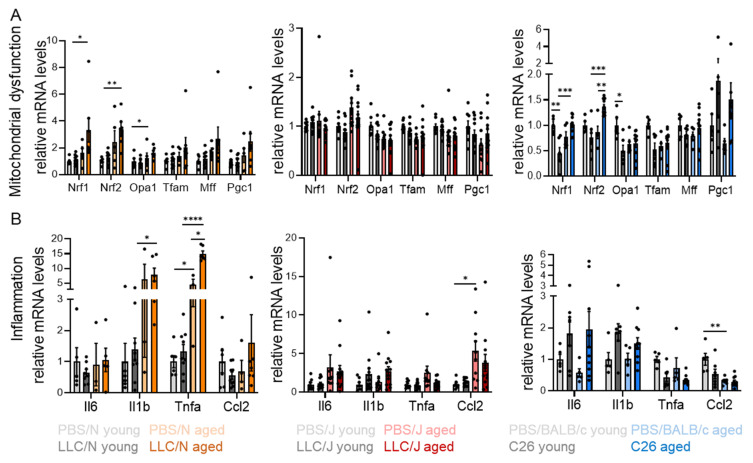
**Age-dependent mitochondrial stress and inflammation in adipose tissue.** (**A**,**B**) Expression of genes involved in different inflammatory and stress-related pathways was analyzed in epididymal white adipose tissue (eWAT) of the same mice as in Figure 2. (**A**) Gene expression of individual markers of mitochondrial dysfunction. (**B**) Expression of inflammatory marker genes. Data are mean ± s.e.m. Statistical analyses were performed using two-way ANOVA with Tukey’s multiple-comparison post hoc test. * *p* < 0.05, ** *p* < 0.01, *** *p* < 0.001, **** *p* < 0.0001.

We and others have previously determined the essential role of WAT for cachexia development [24,25]. Therefore, we next sought to identify age-dependent molecular determinants of adipose tissue wasting, which may explain the strain-dependent differences in cancer-induced body weight loss. To this end, we measured the expression of marker genes involved in different metabolic and stress-related pathways in WAT of LLC/N, LLC/J, and C26 mice. We observed a significant increase in the gene expression of key enzymes in lipolysis (Atgl, hormone-sensitive lipase Hsl, monoacylglycerol lipase Mgl), in WAT of aged LLC/N and LLC/J but not C26 mice (Supplemental Figure S4). In line, we did not observe a common signature of altered gene expression of markers for mitochondrial stress, inflammation, ER stress, or oxidative stress across all mouse models (Figure 5, Supplemental Figure S4). However, individual markers of mitochondrial dysfunction and inflammation were increased in the presence of a tumor in every model (Figure 5A,B), which was largely aggravated by age. Thus, mitochondrial stress and inflammation likely are important mediators of WAT dysfunction in cachexia affected by age.

### 3.5. Circulating IL6 and GDF15 Levels Are Cachexia Markers in C26 Mice

**Figure 6:**
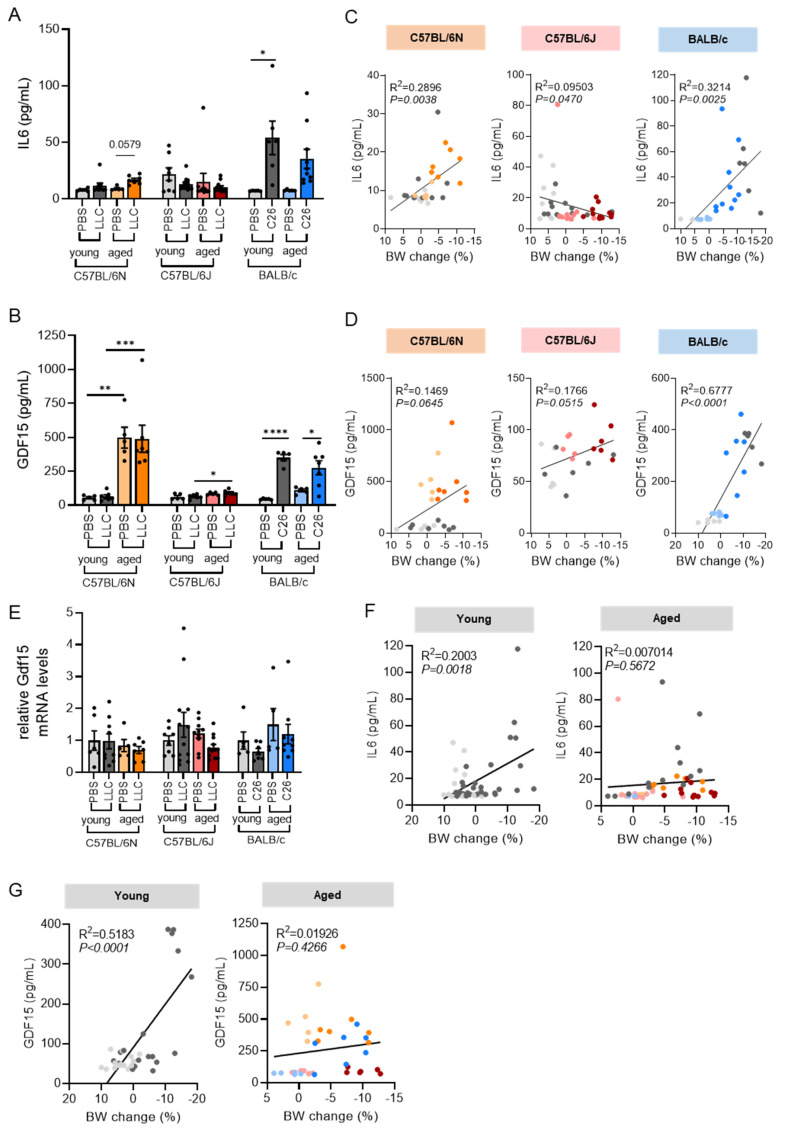
**Circulating IL6 and GDF15 levels are cachexia markers in C26 mice.** (**A**–**G**) Same mice as in Figure 2. (**A**,**B**) Circulating levels of interleukin 6 (IL6) (**A**) and growth differentiation factor 15 (GDF15) (**B**) in young and aged mice. (**C**,**D**) Linear regression analyses comparing plasma IL6 (**C**) or GDF15 (**D**) levels and body weight change (final body weight minus tumor compared to initial body weight). (**E**) Gene expression of GDF15 in murine GC muscles. (**F**,**G**) Linear regression analyses of plasma IL6 (**F**) or GDF15 (**G**) levels and body weight change (final body weight minus tumor compared to initial body weight) in either young or aged mice across all mouse models. Data are mean ± s.e.m. Statistical analyses were performed using two-way ANOVA with Tukey’s multiple-comparison post hoc test (**A**,**B**,**E**), and simple linear regression (**C**,**D**,**F**,**G**). * *p* < 0.05, ** *p* < 0.01, *** *p* < 0.001, **** *p* < 0.0001.

Blood metabolites including low-density lipoprotein (LDL), albumin, total protein, non-esterified fatty acids (NEFA), and glucose were altered by tumor presence or age (Table 1), in line with an altered metabolic state of the animals. As previously observed [26], LDL levels were significantly increased in the presence of tumors in all models, while total cholesterol levels (Table 1) were unaffected by either tumor or age. Circulating glucose levels (Table 1) were significantly decreased by age in all models, and only significantly reduced by the presence of a tumor in young C26 mice. Systemic glucose metabolism (Supplemental Figure S5) was unchanged in BALB/c mice and largely unaffected in C57BL/6 mice of different ages. In line with this, muscle insulin sensitivity (Supplemental Figure S5) was not affected by age in C57BL/6J mice. Upon aging, insulin-like growth factor (IGF1) levels (Table 1) were significantly increased in both C57BL/6 but not BALB/c models. The presence of tumors led to a non-significant reduction of IGF1 in all models, except young LLC/N mice which showed an increase in IGF1 levels upon LLC injection, highlighting again the important substrain differences of C57BL/6 mice. Interleukin 6 (IL6) has long been associated with cachexia development in patients and C26 mice [27]. More recently, also circulating growth/differentiation factor-15 (GDF15) has been identified as a prominent cachexia biomarker correlating with reduced survival in cancer patients [28,29]. We measured circulating IL6 and GDF15 levels in the plasma of LLC/N, LLC/J, and C26 mice. IL6 levels were significantly elevated in young C26 tumor bearing mice, whereas young LLC tumor bearing mice had unaltered IL6 levels. Aged LLC/N and C26 mice showed a non-significant trend towards elevated circulating IL6 (Figure 6A). GDF15 was increased by the presence of a tumor in the C26 model irrespective of age, increased in aged LLC/N and LLC/J mice, and increased in PBS/N mice upon aging (Figure 6B). This indicates that depending on the mouse strain, there are different determinants (age/tumor/interdependence) of GDF15 levels. Indeed, we observed substantial differences between the genetically highly similar C57BL/6J and C57BL/6N substrains in terms of GDF15 levels (as well as circulating glucose and IGF-1) upon ageing (Table 1, Figure 6B). When correlating IL6 and GDF15 levels with the body weight (BW) change across all age groups, we also observed strain differences: IL6 positively correlated with BW loss in C57BL/6N and BALB/c, whereas it correlated negatively with BW loss in C57BL/6J mice (Figure 6C). Since IL6 levels were only increased in mice in which GC mass was significantly altered (aged LLC/N and young C26, see Figure 2C), we hypothesized that IL6 could be a better indicator of muscle loss compared to body weight loss. Interestingly, IL6 levels also significantly correlated with GC muscle mass in C57BL/6N and BALB/c, but not C57BL/6J mice (Supplemental Figure S6). GDF15 only correlated significantly with BW loss in BALB/c mice (Figure 6D). Neither IL6 nor GDF15 therefore seem to be universal cachexia biomarkers in mouse models. Since GDF15 levels were elevated in aged LLC/N and LLC/J mice, but not aged C26 mice, GDF15 increases occurred parallel to worsened body weight loss upon ageing (Figure 2), indicating that it may play a role in this phenotype. Of note, Gdf15 mRNA levels in muscle were unchanged between all groups (Figure 6E).

**Figure 7:**
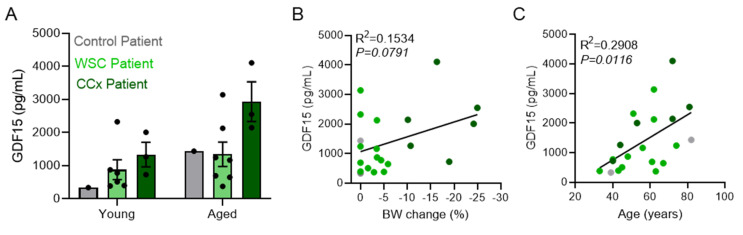
**GDF15 significantly correlates with age of patients, but not body weight (BW) change.** We compared GDF15 levels of control patients without tumor, as well as patients with gastrointestinal cancer with or without BW loss. (**A**) Total plasma GDF15 levels. (**B**,**C**) Linear regression analysis comparing human plasma GDF15 levels and BW change in percentage (**B**) or age in years (**C**) of patients. Data are mean ± s.e.m. Statistical analyses were performed using two-way ANOVA with Tukey’s multiple-comparison *post hoc* test (**A**), and simple linear regression (**B**,**C**).

In patients, we observed a trend to elevated circulating GDF15 levels in weight losing (Ccx) vs. weight stable cancer (WSC) patients (Figure 7A). In the aged group, we observed the same regulation as in the young group, but on an overall higher level. GDF15 levels correlated significantly with age, but not weight loss, in these patients (Figure 7B,C), in line with an important role of GDF15 in ageing, as suggested by the LLC/N data. Interestingly, when assessing the correlations between body weight loss and cytokine levels between all mouse models, we observed a significant positive correlation between IL6 or GDF15 and weight loss only in young animals. These correlations were not present in aged animals (Figure 6F,G). This phenomenon was also apparent when correlating weight loss and cytokine levels in BMI- and weight loss matched patient groups with healthy, cachectic, and non-cachectic patients with cancer: a significant positive correlation between circulating cytokine levels (IL6, IL1β, IL10) and body weight loss was observed only in the young patient population (≤55 years) (Table 2), although weight loss was comparable between groups (see patient characteristics in Table 3).

## 4. Discussion

We here describe for the first time the influence of age across different mouse models of cancer cachexia, a disease that is mostly linked to old age in humans. Importantly, we find significant strain- and tumor-type specific differences regarding the degree of weight loss, tissue loss, molecular markers, and circulating mediators. Age did not influence C26-mediated induction or progression of weight or tissue loss in the BALB/c mouse, while it severely impacted on weight loss in the C57BL/6 mouse strains. Indeed, only aged C57BL/6N mice developed weight loss upon LLC injection, while the tumor had little effect on the body weight of young animals of the same strain. In C57BL/6J mice, aging aggravated weight loss. These effects were largely recapitulated by the expression of atrophy and stress markers in muscle and adipose tissue. Tumors induced a reduction in the fiber cross-sectional area in all strains, but fibers were smallest in aged LLC-injected animals. We have focused exclusively on gastrocnemius muscle; however, it will be important for future studies to also include data on other muscle types. Our data are in line with a previous report showing no age effect in the C26 model [20], but expand our current knowledge by the additional description of two further models.

Our study provides the first evidence that C57BL/6J and C57BL/6N mice are not identical in terms of LLC-induced weight loss, expression of atrophy markers in muscle and adipose tissue, and circulating cachexia markers. As such, it presents an important reference point for many studies investigating cancer cachexia in genetically modified mice (where the exact background of the mice is often unclear, or mice are of mixed C57BL/6J/C57BL/6N background). A recent study comparing C57BL/6J and C57BL/6N on a large scale across different centers [10] has found not only considerable genetic but also phenotypic differences between the two strains. For instance, oxygen consumption, energy expenditure, and body composition were subject to strain differences, as was grip strength—important readouts for cachexia experiments. Of note, genetic, behavioral, and metabolic differences between closely related substrains are not limited to the C57BL/6 background but have also been described for other backgrounds, including the BALB/c. In that regard, genetically highly similar BALB/c substrains show variations in their physical activity [30], which may also affect cachexia development. This should be taken into consideration when designing experiments using knockout mice in the future, and underlines the importance of using littermates as control animals in all studies [31].

A differentiation between cancer-induced cachexia and age-induced sarcopenia in subjects with higher age is not always possible. Our study has used mice aged 15–20 months, so before the onset of considerable loss of body and muscle weight associated with aging [32]. Atrogin1 and MuRF1 are atrogenes typically associated with muscle wasting, but not with age-related sarcopenia [33]. In line with this, we did not observe an altered expression of these factors in relation to aging, but only in relation to tumor cell-induced loss of muscle mass (Figure 4). Ubiquitin-proteasome proteolysis is therefore a pathway more relevant for cachexia than sarcopenia. Interestingly, Cdkn1a expression followed the same pattern as Atrogin1 and MuRF1. Cdkn1a (p21) was recently identified as a gene most robustly upregulated in cachectic muscle when integrating transcriptome datasets across five different cachexia models [34]. Apart from the above-mentioned effects on protein metabolism, Cdkn1a inhibits cell cycle and induces senescence, and was shown to be sufficient to induce muscle atrophy [21]. Our data further underline the importance of muscle Cdkn1a as an atrogene.

Inflammation is a central hallmark of cachexia [1]. We have therefore assessed inflammatory cytokines, spleen and lymph node mass as well as the CD4/CD8 T cell ratio in aged and young cachectic animals. An age-dependent association between the CD4/CD8 ratio and frailty has been noted in patients with HIV, which are also prone to develop cachexia [16]; however, this seemed not to be the case in our mouse models of cancer cachexia. In addition, tumor vascularization and endothelial permeability are tightly linked with inflammation [35] and may influence circulating cachexia mediators, yet the expression of vascularization marker genes (Epas1, Tie2, Vegfr2, Pecam1 (CD31) [36] was not different between young and aged mice across all experiments (data not shown).

IL6 and GDF15 are important mediators of cancer cachexia with therapeutic relevance, since blocking either IL6 or GDF15 resulted in improved disease outcomes in cachectic animals [28,37]. Importantly, both factors are also elevated in aged individuals and linked to unfavorable outcomes and frailty [38,39]. Gdf15 was shown to be expressed in skeletal muscle under different stress conditions [40,41]. However, these studies were inconsistent in the role of skeletal muscle in GDF15 secretion, with one study showing muscle-secreted GDF15 as regulator of adipose lipolysis, while the other concluded that skeletal muscle GDF15 did not enter circulation in mice but rather exerted local effects. We observed an age-dependent increase in circulating GDF15 levels but no alterations to Gdf15 expression in skeletal muscle, indicating that other sources of GDF15 are more important in regulating circulating GDF15 in cachexia in our models. Plasma GDF15 levels also tended to be higher in cachectic patients, and more so upon aging. The increase in GDF15 upon cachexia was much more evident in BALB/c compared to C57BL/6 mice. Similarly, high circulating IL6 levels described as classical cachexia feature [42] were absent in the LLC models, despite apparent weight loss (Figure 6). Our study thus demonstrates that a hint of caution is warranted in selecting mouse models for cancer cachexia, as overrepresentation of certain circulating factors such as IL6 may occur when exclusively using the C26 mouse model. BALB/c mice have been shown to mount an exaggerated systemic inflammation as assessed by increased plasma and hepatic cytokine levels [43]. Importantly, tissue-intrinsic pathways related to cachexia such as atrophy and metabolic stress pathways are equally regulated across all models despite individual differences to the gene expression pattern within these pathways (Figure 4 and Figure 5, Supplementary Figures S2 and S3). To increase the translatability of animal studies to clinical practice, we should therefore consider analyzing cachexia parameters in different mouse models and different mouse strains [26]. The C26 mouse model seems unaltered by aging, underlining its importance and relevance as cancer cachexia model for preclinical studies. Of note, we only included male mice in our study, and further investigations are necessary to define the interrelation between age, strain, and sex.

Lastly, it is important to note that we also observed an age-effect in our patient cohort. Despite the small sample size (*n* = 14/group), we demonstrated significant correlations between circulating cytokines and weight loss, in line with their important function as cachexia biomarkers [44]. However, these correlations were lost in the aged patient group. More studies investigating age differences in cachexia are needed in order to address the dynamic regulation of wasting metabolism and inflammatory mediators in aging.

## 5. Conclusions

We have shown that aging has a substantial effect on cachexia development depending on the mouse model studied. This has important implications for the design of future studies on cancer cachexia in mice. In patients, cachexia biomarkers can have a different prognostic value dependent on the age of the subjects. The general mechanistic principles underlying tissue wasting in cachexia are unaffected by mouse strain and age.

## Figures and Tables

**Table 1:** **Blood metabolites in mice are altered by tumor presence and age.** Plasma levels of cholesterol, low-density lipoprotein (LDL), non-esterified fatty acids (NEFA), glucose, albumin, total protein, and insulin-like growth factor (IGF-1) in three mouse models of cachexia with groups of different age as explained in Figure 2. Data are mean ± s.e.m. Statistical analyses were performed using two-way ANOVA with Tukey’s multiple-comparison post hoc test. * *p* < 0.05, ** *p* < 0.01, *** *p* < 0.001, **** *p* < 0.0001 compared to PBS mice. ^#^ *p* < 0.05, ^##^ *p* < 0.01, ^###^ *p* < 0.001, ^#####^ *p* < 0.0001 comparing age.

Experiment	n	Cholesterol (mg/dL)	LDL (mg/dL)	NEFA (mmol/L)	Glucose (mg/dL)	Albumin (g/L)	Total Protein (g/L)	n	IGF-1 (ng/mL)
**PBS/N young**	6	80.67 ± 2.99	9.53 ± 0.36	0.42 ± 0.04	319.5 ± 16.85	25.74 ± 0.35	45.37 ± 0.65	3	379.16 ± 28.45
**LLC/N young**	9	93.67 ± 3.80	21.37 ± 1.58 ****	0.30 ± 0.04	288.7 ± 9.472	21.75 ± 0.82 **	42.80 ± 1.31	9	292.10 ± 20.64
**PBS/N aged**	5	88.00 ± 2.30	17.80 ± 0.37 ^##^	0.27 ± 0.07	213.0 ± 11.34 ^####^	24.19 ± 0.55	48.93 ± 1.15	5	664.42 ± 21.50 ^###^
**LLC/N aged**	7	85.71 ± 3.91	22.56 ± 1.93	0.19 ± 0.03	185.0 ± 10.75 ^####^	23.03 ± 0.78	47.10 ± 1.35	7	526.10 ± 43.70 *^####^
**PBS/J young**	8	69.38 ± 2.23	11.84 ± 0.95	0.36 ± 0.03	309.1 ± 18.62	21.82 ± 0.39	40.02 ± 0.51	5	236.88 ± 24.11
**LLC/J young**	12	82.25 ± 3.38	18.04 ± 1.33 **	0.41 ± 0.03	356.2 ± 20.91	21.98 ± 0.44	41.56 ± 0.91	6	290.43 ± 11.38
**PBS/J aged**	10	76.20 ± 5.89	11.18 ± 0.51	0.15 ± 0.02 ^####^	269.8 ± 16.15	24.84 ± 0.88 ^#^	46.48 ± 1.04 ^###^	5	322.30 ± 18.08 ^#^
**LLC/J aged**	12	85.08 ± 4.20	20.08 ± 1.26 ****	0.26 ± 0.02 ^##^	279.1 ± 14.96 ^#^	21.12 ± 0.53 ***	42.15 ± 0.87 **	6	305.80 ± 10.53
**PBS/BALBc young**	5	109.60 ± 3.43	8.84 ± 0.34	0.30 ± 0.10	325.60 ± 15.69	21.05 ± 0.51	41.49 ± 2.19	5	459.30 ± 34.02
**C26 young**	6	123.50 ± 4.01	18.85 ± 1.34 ****	0.14 ± 0.03	220.00 ± 11.69 ***	22.68 ± 0.44	52.27 ± 0.90 ****	5	389.18 ± 18.50
**PBS/BALBc aged**	5	126.80 ± 4.04	12.36 ± 0.52	0.22 ± 0.02	209.00 ± 17.641 ^###^	22.52 ± 0.76	43.68 ± 0.99	5	510.96 ± 25.08
**C26 aged**	10	125.50 ± 4.68	18.64 ± 0.93 ***	0.18 ± 0.01	184.80 ± 11.38	20.83 ± 0.42	46.88 ± 0.87 ^#^	7	475.60 ± 25.79

**Table 2:** **Significant correlation between cytokine levels and weight loss only in young patients.** Correlations of human plasma cytokine and metabolite levels with body weight change of control patients without cancer, and patients with gastrointestinal cancers with and without body weight loss. Numbers of tested patients are included in the table. R^2^ and significance were calculated using simple linear regression analysis and significant changes are highlighted in orange.

	Young (≤55y)			Aged (>55y)		
Plasma	n	R^2^	*p* Value	n	R^2^	*p* Value
IFNγ	14	0.01020	0.7312	14	0.004835	0.8133
IL10	14	0.5571	0.0022	14	0.003069	0.8508
IL13	14	0.1103	0.2461	14	0.04233	0.4804
IL1b	14	0.4384	0.0099	14	0.006826	0.7789
IL6	14	0.4499	0.0086	14	0.2428	0.0734
IL8	14	0.02732	0.5722	14	0.006336	0.7868
MCP1	14	0.2092	0.1001	14	0.04584	0.4623
MIP1A	14	0.001337	0.9012	14	6.88 × 10^−7^	0.9978
MIP1B	14	0.1588	0.1581	14	0.02744	0.5886
Tnfa	14	0.1056	0.2570	14	0.01546	0.6719
Hemoglobin	14	0.2449	0.1019	14	0.4042	0.0355
CRP	14	0.09554	0.2822	14	0.06038	0.3971
Albumin	14	0.1068	0.2542	14	0.1586	0.1584
Cholesterol	13	0.1093	0.2698	14	0.02556	0.5851
HDL	14	0.1124	0.2413	14	0.05848	0.4049
LDL	14	0.01040	0.7286	14	0.02746	0.5713
Triglycerides	14	0.06374	0.3839	14	0.1144	0.2369

**Table 3:** **Human clinical data.** BMI: body mass index; BW: body weight. Data are mean ± s.e.m. Statistical analyses were performed with unpaired *t*-test or Mann-Whitney test.

Clinical Parameters	Young Patients (≤55 years)	Aged Patients (>55 years)	*p* Value
Overall (*n*)	14	14	
Female/Male (*n*)	7/7	8/6	
Age (years)	44.86 ± 1.565	68.21 ± 2.204	<0.0001
BW loss (%)	−8.010 ± 2.602	−7.171 ± 2.216	0.8080
BMI (kg/m^2^)	24.51 ± 1.173	23.56 ± 1.016	0.5432

## Data Availability

The datasets used for the current study are available from the corresponding author on reasonable request.

## Supplementary Materials

The following supporting information can be downloaded at: https://www.mdpi.com/article/10.3390/cancers14010090/s1. Figure S1: Initial body weight is unchanged between PBS and tumor cell-injected mice of similar age. Figure S2: Age-dependent alterations of immune cell subtypes are unlikely to drive differences in cachexia development. Figure S3: Genes related to mitochondrial dysfunction, autophagy, ER stress, oxidative stress and apoptosis are not commonly altered upon cachexia or aging in gastrocnemius muscles (GC) of different mouse strains. Figure S4: Genes related to metabolism, ER stress, and oxidative stress are differentially altered upon cachexia and aging in epididymal white adipose tissue (eWAT) of different cachexia mouse models. Figure S5: Glucose tolerance and insulin sensitivity are largely unaffected by either age or tumor in different cachexia mouse models. Figure S6: Correlation between IL6 and GC muscle mass.
