# Supplementary material for: Aging Aggravates Cachexia in Tumor-Bearing Mice
Source: Cancers (Basel). 2021 Dec 24;14(1):90. doi: 10.3390/cancers14010090 (PMC8750471; doi:10.3390/cancers14010090)
Supplement: Supplementary file 1 [file cancers-14-00090-s001.zip › 1450699_supplemental figures_proofread_JG.pptx]

## Slide 1
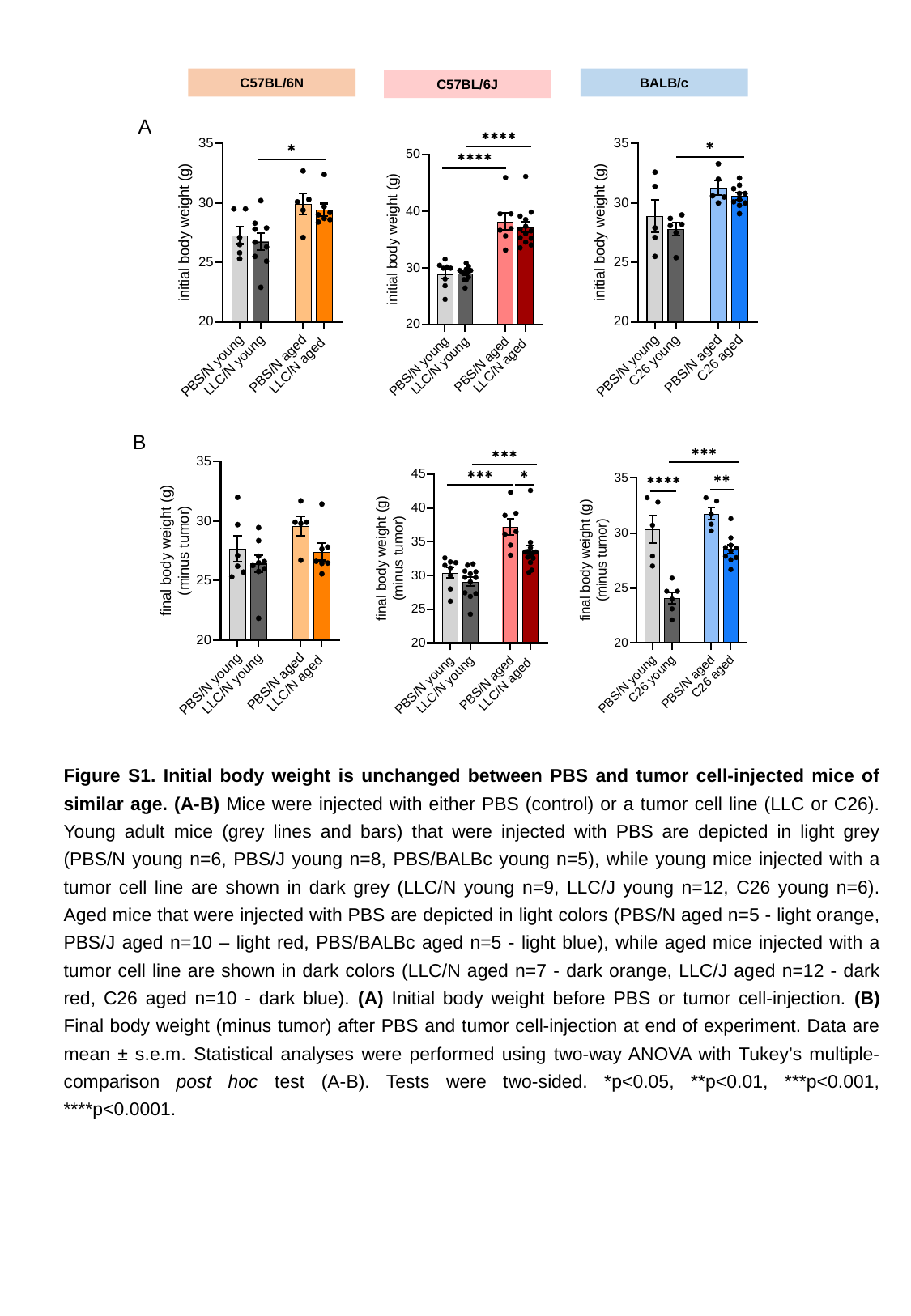

C57BL/6N
BALB/c
C57BL/6J
A
B
Figure S1. Initial body weight is unchanged between PBS and tumor cell-injected mice of similar age. (A-B) Mice were injected with either PBS (control) or a tumor cell line (LLC or C26). Young adult mice (grey lines and bars) that were injected with PBS are depicted in light grey (PBS/N young n=6, PBS/J young n=8, PBS/BALBc young n=5), while young mice injected with a tumor cell line are shown in dark grey (LLC/N young n=9, LLC/J young n=12, C26 young n=6). Aged mice that were injected with PBS are depicted in light colors (PBS/N aged n=5 - light orange, PBS/J aged n=10 – light red, PBS/BALBc aged n=5 - light blue), while aged mice injected with a tumor cell line are shown in dark colors (LLC/N aged n=7 - dark orange, LLC/J aged n=12 - dark red, C26 aged n=10 - dark blue). (A) Initial body weight before PBS or tumor cell-injection. (B) Final body weight (minus tumor) after PBS and tumor cell-injection at end of experiment. Data are mean ± s.e.m. Statistical analyses were performed using two-way ANOVA with Tukey’s multiple-comparison post hoc test (A-B). Tests were two-sided. *p<0.05, **p<0.01, ***p<0.001, ****p<0.0001.

## Slide 2
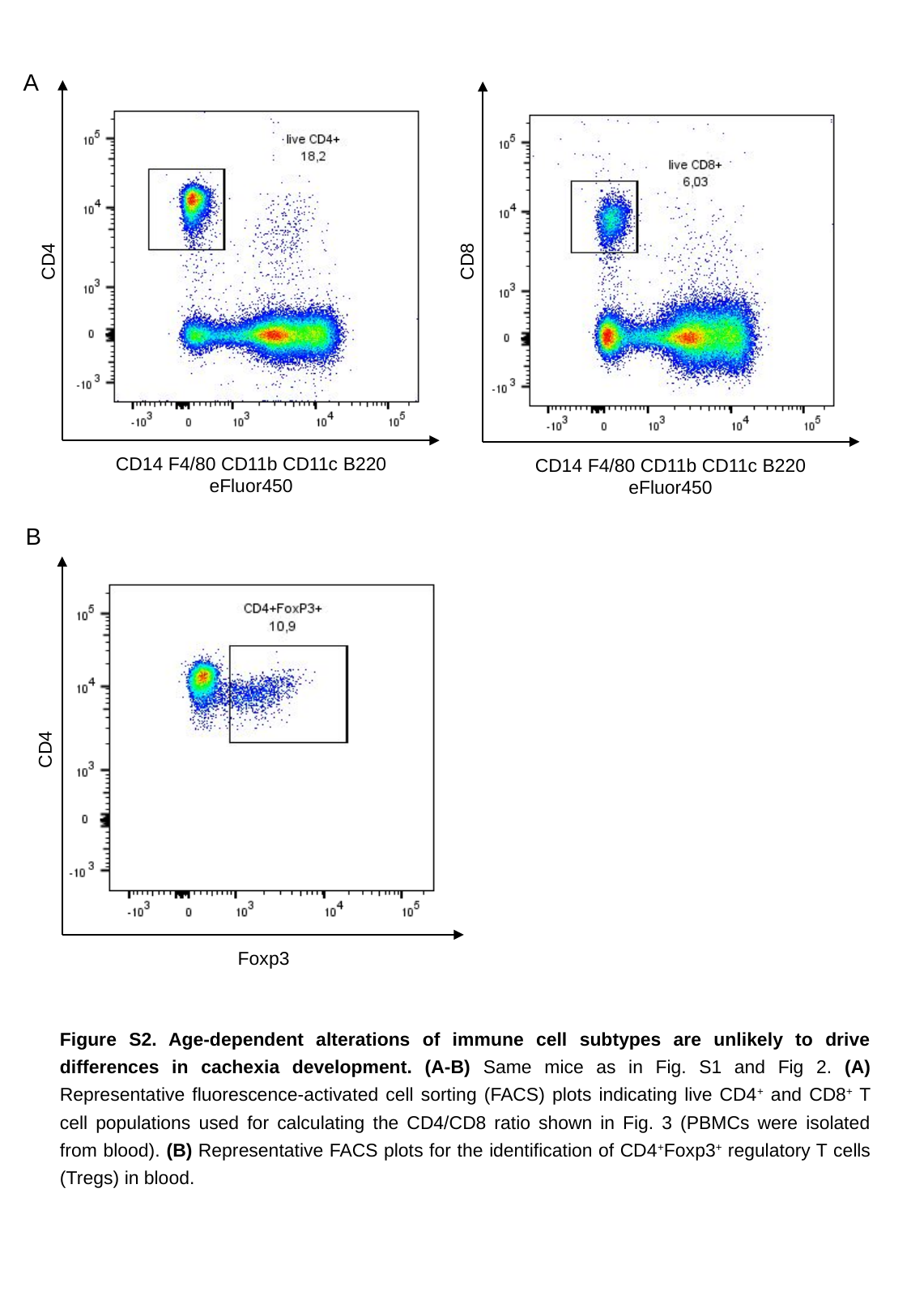

A
CD4
CD14 F4/80 CD11b CD11c B220 eFluor450
CD8
CD14 F4/80 CD11b CD11c B220 eFluor450
B
CD4
Foxp3
Figure S2. Age-dependent alterations of immune cell subtypes are unlikely to drive differences in cachexia development. (A-B) Same mice as in Fig. S1 and Fig 2. (A) Representative fluorescence-activated cell sorting (FACS) plots indicating live CD4+ and CD8+ T cell populations used for calculating the CD4/CD8 ratio shown in Fig. 3 (PBMCs were isolated from blood). (B) Representative FACS plots for the identification of CD4+Foxp3+ regulatory T cells (Tregs) in blood.

## Slide 3
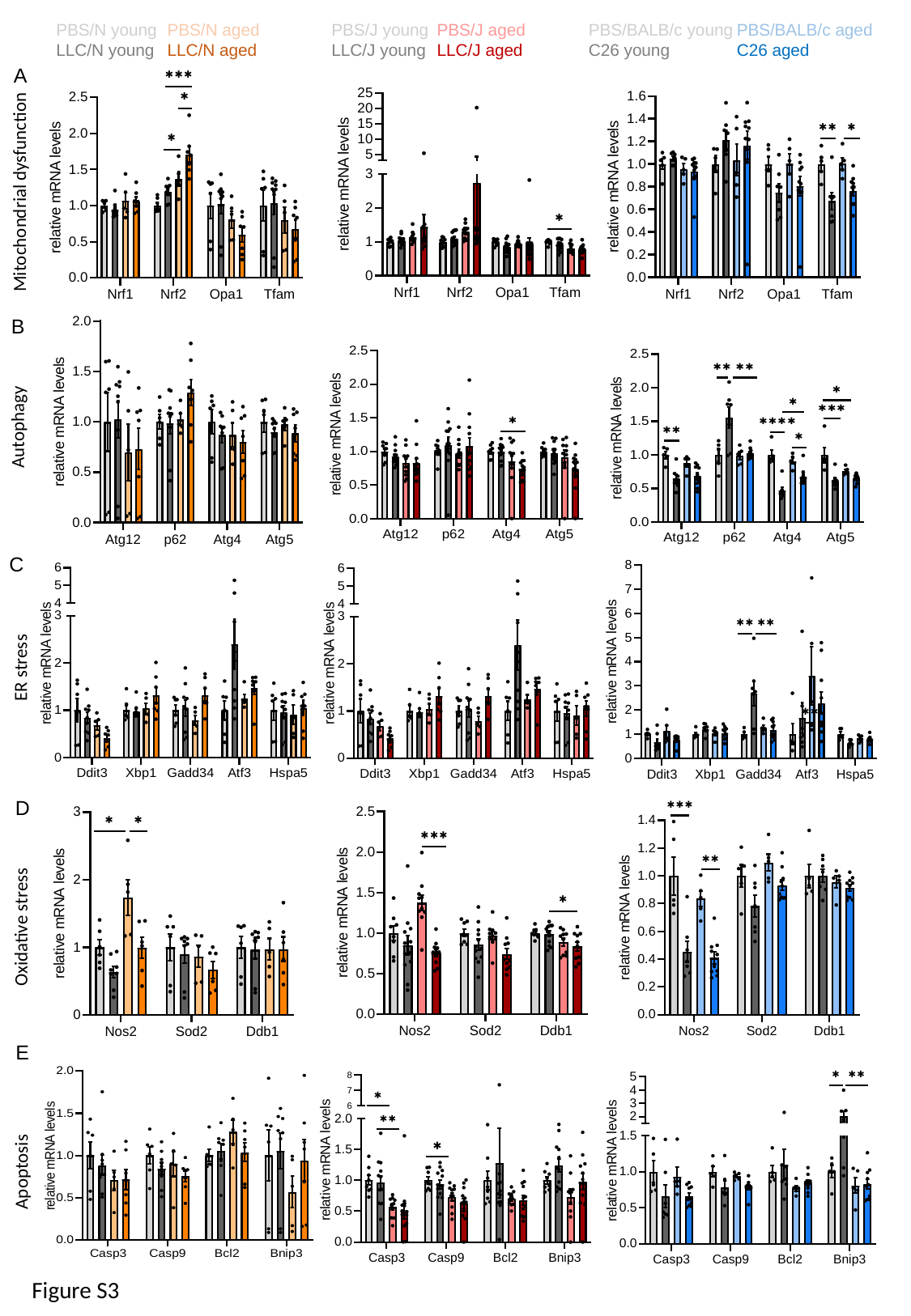

PBS/N young
LLC/N young
PBS/N aged
LLC/N aged
PBS/J young
LLC/J young
PBS/J aged
LLC/J aged
PBS/BALB/c young
C26 young
PBS/BALB/c aged
C26 aged
A
Mitochondrial dysfunction
B
Autophagy
C
ER stress
D
Oxidative stress
E
Apoptosis
Figure S3

## Slide 4
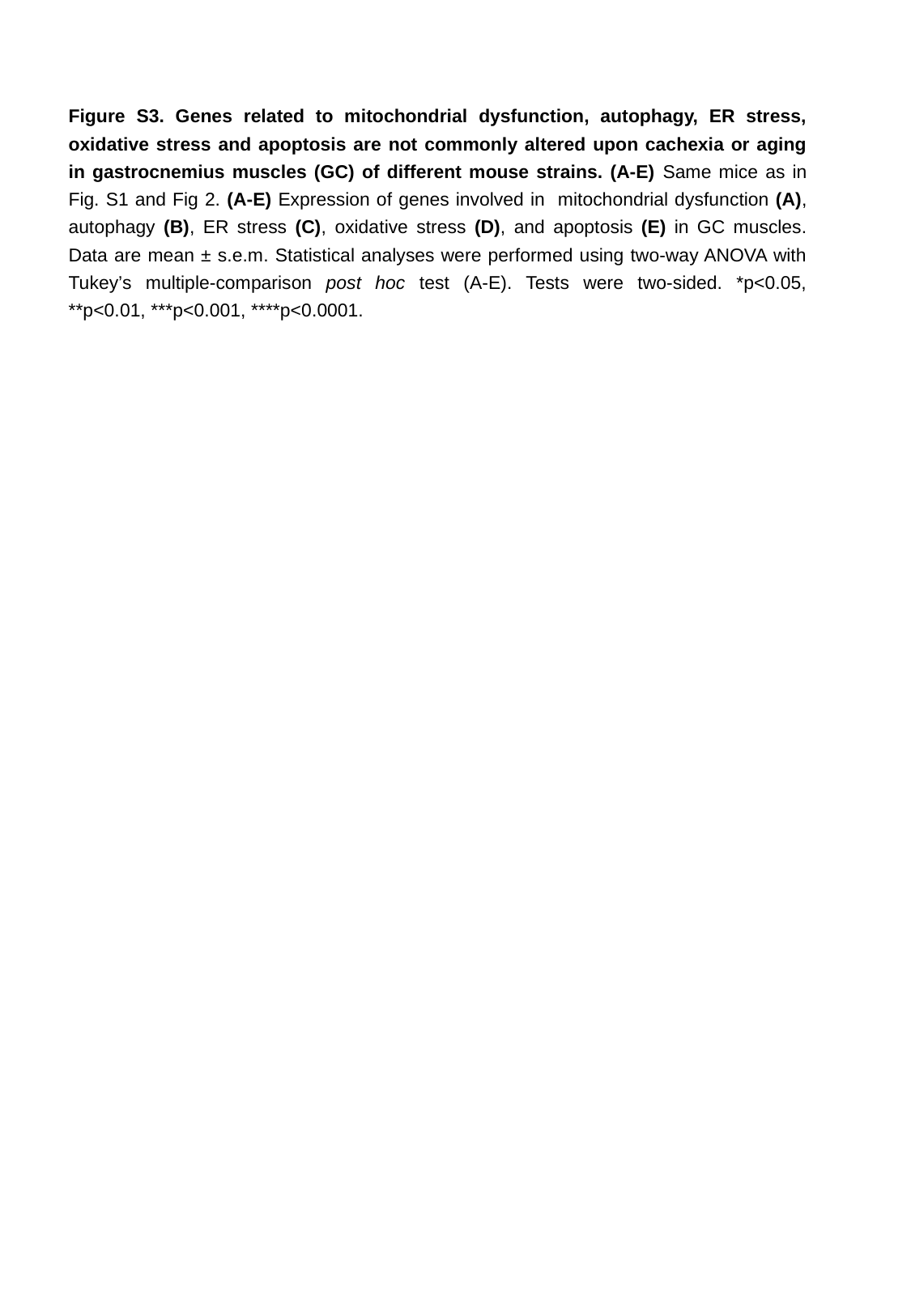

Figure S3. Genes related to mitochondrial dysfunction, autophagy, ER stress, oxidative stress and apoptosis are not commonly altered upon cachexia or aging in gastrocnemius muscles (GC) of different mouse strains. (A-E) Same mice as in Fig. S1 and Fig 2. (A-E) Expression of genes involved in mitochondrial dysfunction (A), autophagy (B), ER stress (C), oxidative stress (D), and apoptosis (E) in GC muscles. Data are mean ± s.e.m. Statistical analyses were performed using two-way ANOVA with Tukey’s multiple-comparison post hoc test (A-E). Tests were two-sided. *p<0.05, **p<0.01, ***p<0.001, ****p<0.0001.

## Slide 5
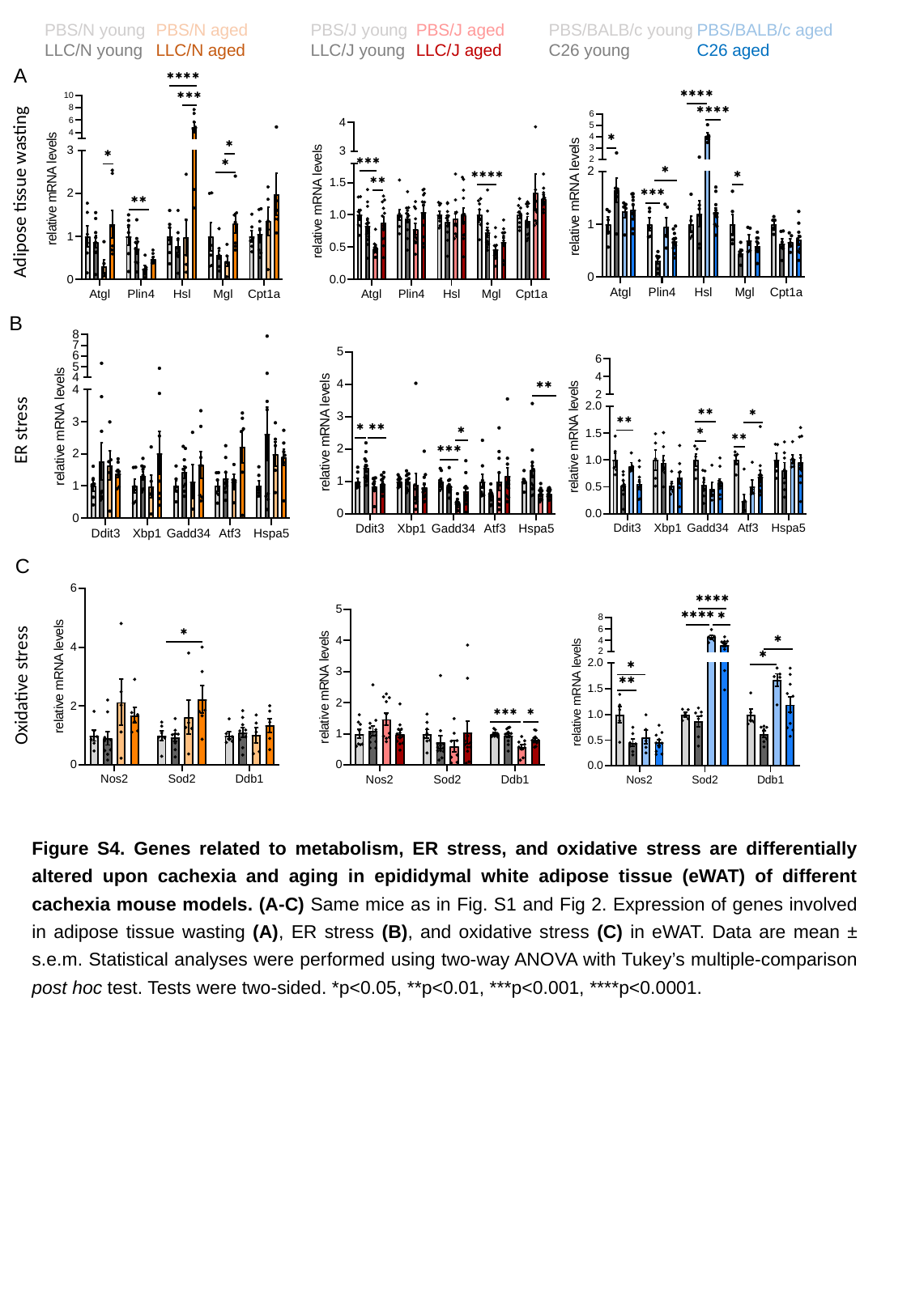

PBS/N young
LLC/N young
PBS/N aged
LLC/N aged
PBS/J young
LLC/J young
PBS/J aged
LLC/J aged
PBS/BALB/c young
C26 young
PBS/BALB/c aged
C26 aged
A
Adipose tissue wasting
B
ER stress
C
Oxidative stress
Figure S4. Genes related to metabolism, ER stress, and oxidative stress are differentially altered upon cachexia and aging in epididymal white adipose tissue (eWAT) of different cachexia mouse models. (A-C) Same mice as in Fig. S1 and Fig 2. Expression of genes involved in adipose tissue wasting (A), ER stress (B), and oxidative stress (C) in eWAT. Data are mean ± s.e.m. Statistical analyses were performed using two-way ANOVA with Tukey’s multiple-comparison post hoc test. Tests were two-sided. *p<0.05, **p<0.01, ***p<0.001, ****p<0.0001.

## Slide 6
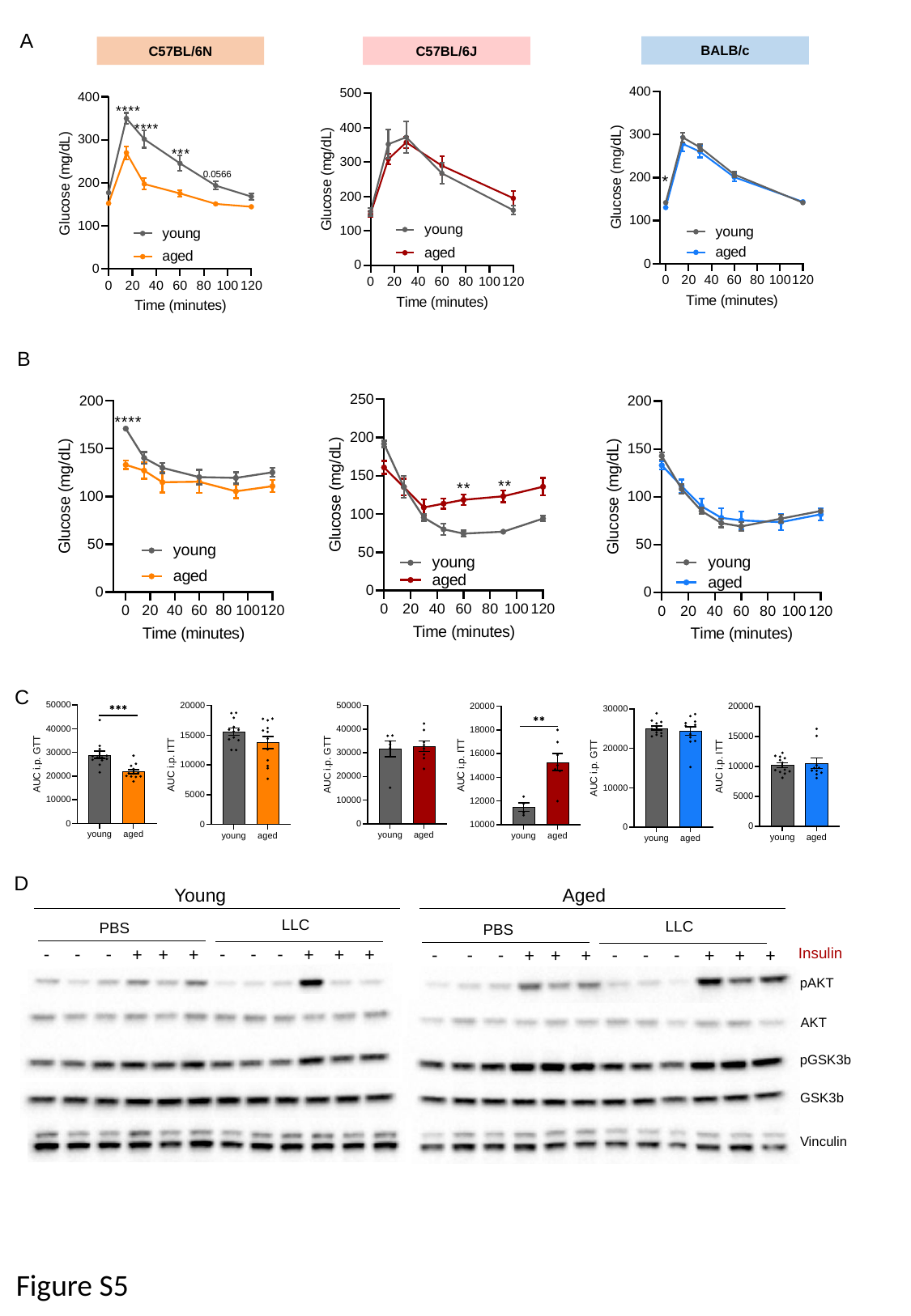

A
BALB/c
C57BL/6N
C57BL/6J
B
C
D
Young
Aged
LLC
LLC
PBS
PBS
- - - + + + - - - + + +
pAKT
AKT
pGSK3b
GSK3b
Vinculin
- - - + + + - - - + + +
Insulin
Figure S5

## Slide 7
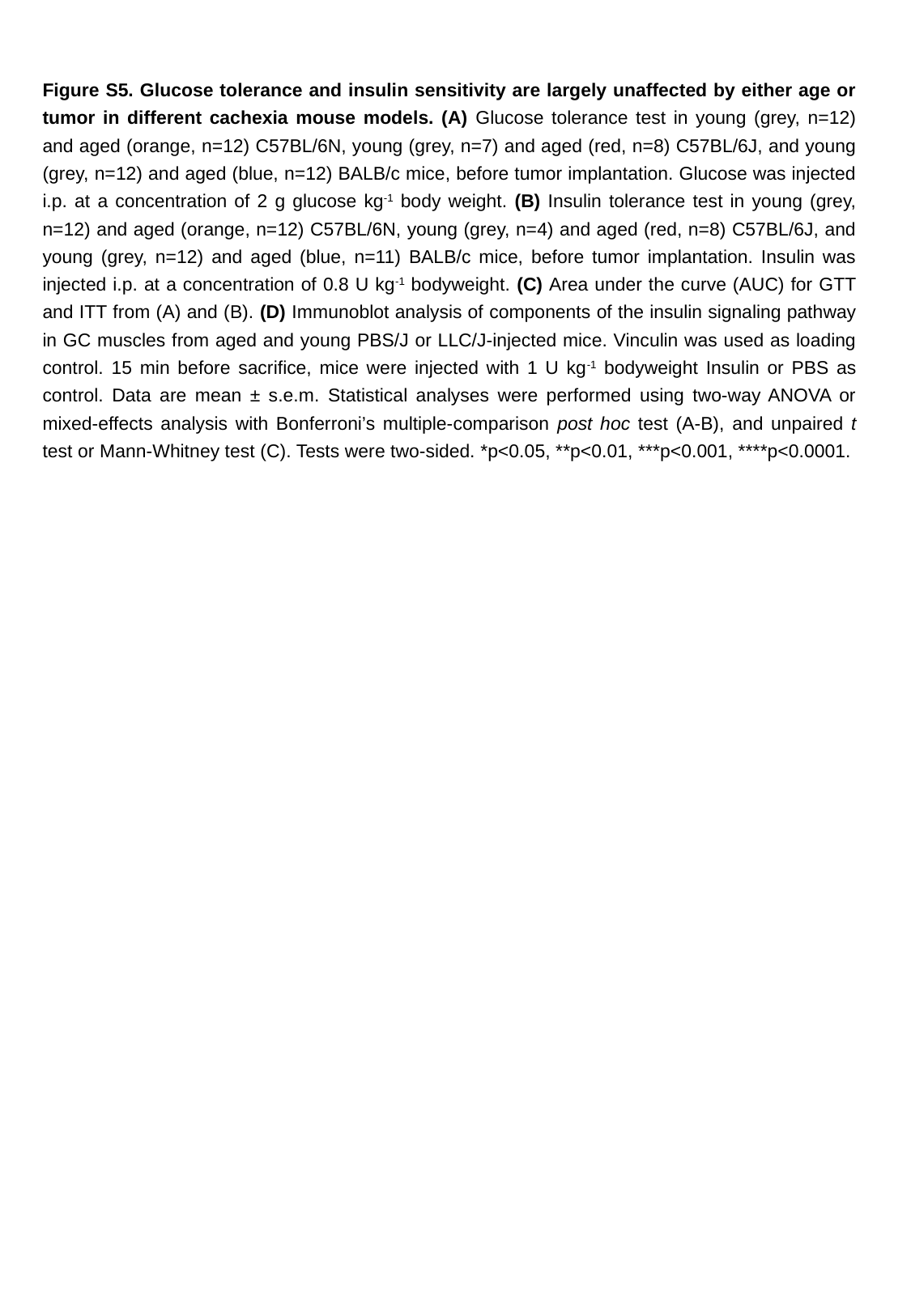

Figure S5. Glucose tolerance and insulin sensitivity are largely unaffected by either age or tumor in different cachexia mouse models. (A) Glucose tolerance test in young (grey, n=12) and aged (orange, n=12) C57BL/6N, young (grey, n=7) and aged (red, n=8) C57BL/6J, and young (grey, n=12) and aged (blue, n=12) BALB/c mice, before tumor implantation. Glucose was injected i.p. at a concentration of 2 g glucose kg-1 body weight. (B) Insulin tolerance test in young (grey, n=12) and aged (orange, n=12) C57BL/6N, young (grey, n=4) and aged (red, n=8) C57BL/6J, and young (grey, n=12) and aged (blue, n=11) BALB/c mice, before tumor implantation. Insulin was injected i.p. at a concentration of 0.8 U kg-1 bodyweight. (C) Area under the curve (AUC) for GTT and ITT from (A) and (B). (D) Immunoblot analysis of components of the insulin signaling pathway in GC muscles from aged and young PBS/J or LLC/J-injected mice. Vinculin was used as loading control. 15 min before sacrifice, mice were injected with 1 U kg-1 bodyweight Insulin or PBS as control. Data are mean ± s.e.m. Statistical analyses were performed using two-way ANOVA or mixed-effects analysis with Bonferroni’s multiple-comparison post hoc test (A-B), and unpaired t test or Mann-Whitney test (C). Tests were two-sided. *p<0.05, **p<0.01, ***p<0.001, ****p<0.0001.

## Slide 8
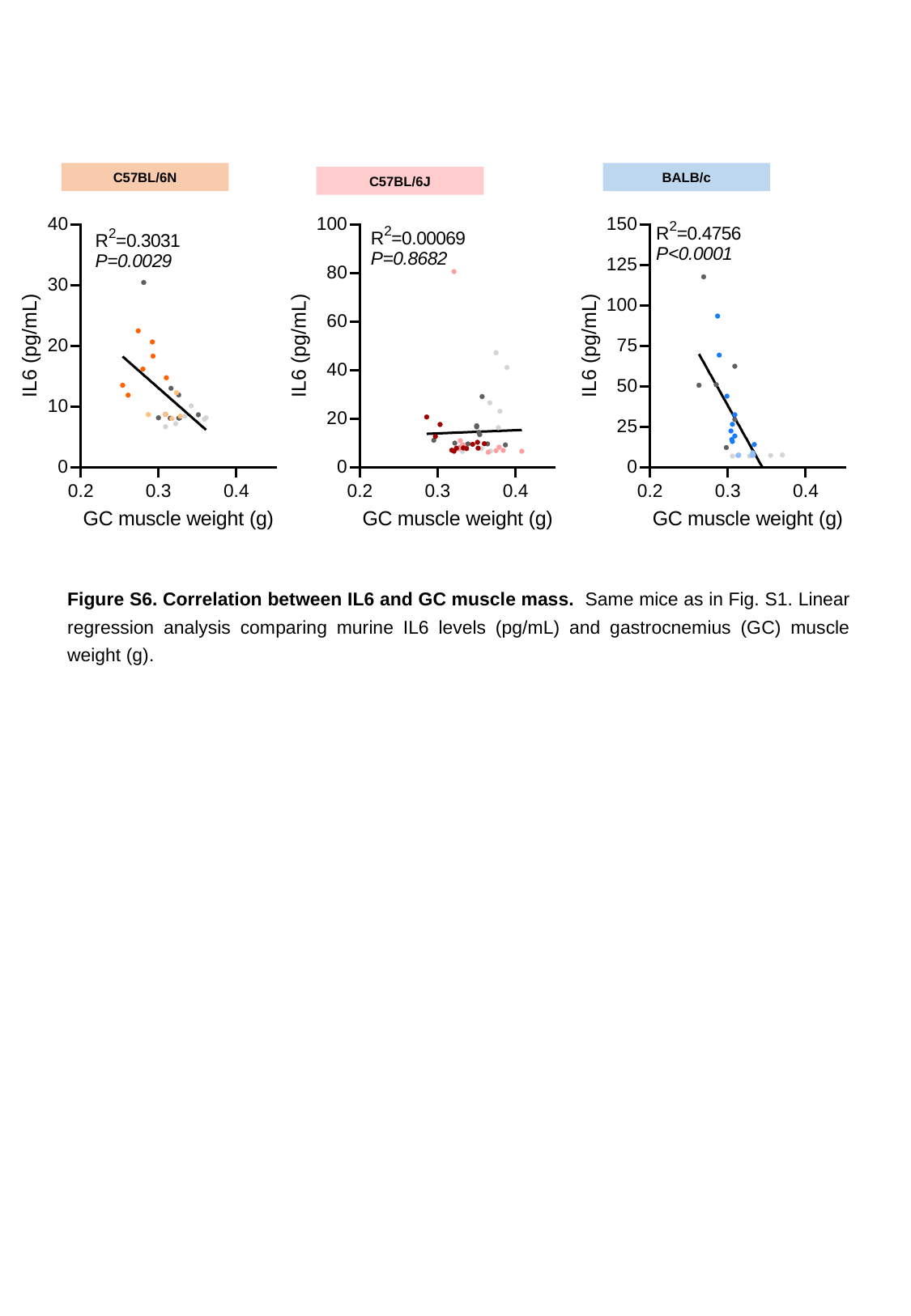

C57BL/6N
BALB/c
C57BL/6J
Figure S6. Correlation between IL6 and GC muscle mass. Same mice as in Fig. S1. Linear regression analysis comparing murine IL6 levels (pg/mL) and gastrocnemius (GC) muscle weight (g).
